# Supplementary material for: Preparative Fractionation of Brazilian Red Propolis Extract Using Step-Gradient Counter-Current Chromatography
Source: Molecules. 2024 Jun 9;29(12):2757. doi: 10.3390/molecules29122757 (PMC11206579; doi:10.3390/molecules29122757)
Supplement: Supplementary file 1 [file molecules-29-02757-s001.zip › molecules-3019104-supplementary.pdf]

Supplementary material

Preparative fractionation of Brazilian red propolis extract by step-gradient counter-current chromatography, Begoña Gimenez-Cassina Lopez, et al, Molecules, 2024

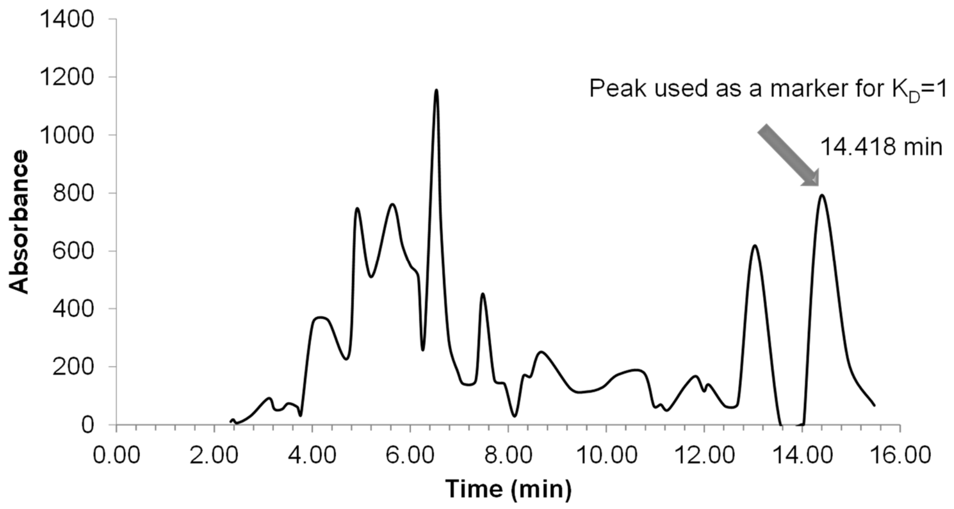

Figure S1. HPLC-UV chromatogram of the red propolis ethanolic extract.

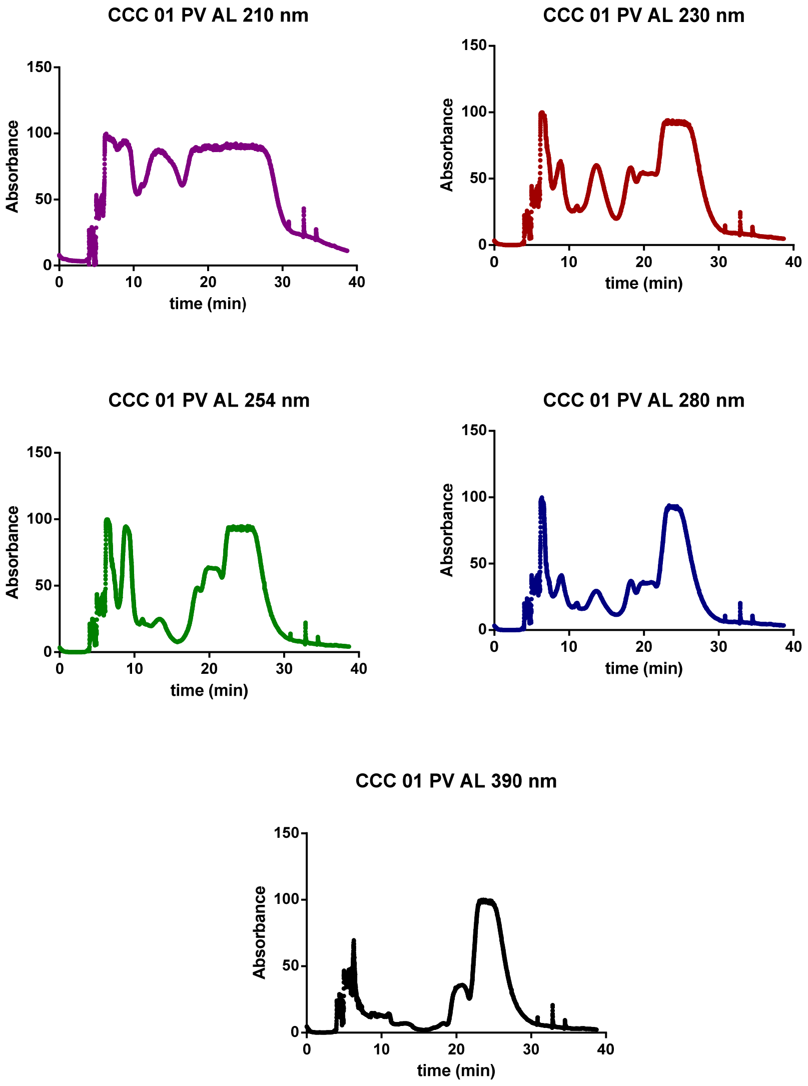

Figure S2. CCC chromatograms at different wavelengths. Conditions: Mini 17.4 mL column, 0 – 25 min HMWat (5:4:1 v/v/v), NP mode, 1 mL.min^{-1}, 2100 rpm, 40 mg of crude made in lower phase.

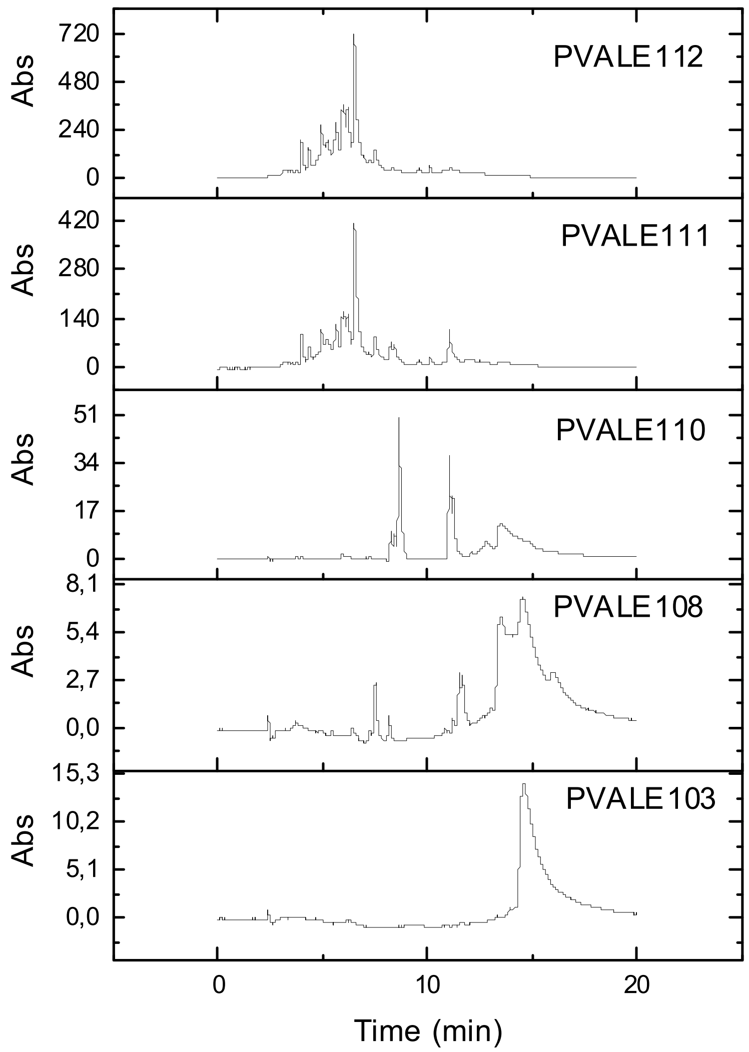

Figure S3. HPLC analysis of CCC elution fractions from Figure S2.

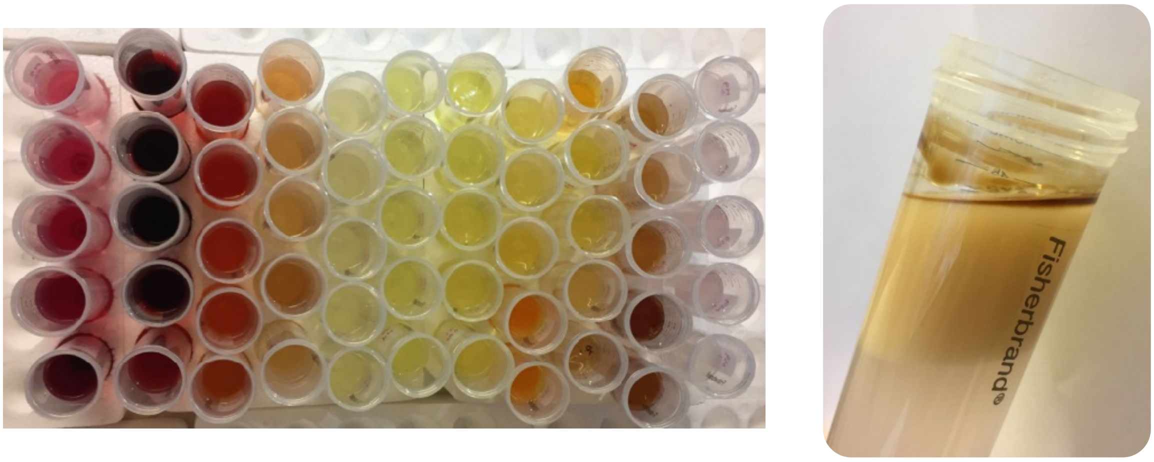

(A) (B)

Figure S4. Image of representative CCC Midi fractions of red propolis separation: (A) elution stage only, starting from the bottom right (nonpolar compounds) and following serpentine path; (B) nonpolar fraction containing waxes (top of the tube) eluting with the solvent front.

NMR Assignments

The structure of the 2′-hydroxy-4′,7-dimethoxyisoflavan with the numbering of atoms is displayed in Figure 4. We consulted the literature to assess the structure of the 2′-hydroxy-4′,7-dimethoxyisoflavan and comparison with other structures of this family of isoflavan [12]. Its ^{1}H NMR spectrum (500 MHz; δ_H in ppm; $CDCl_3$) (Table S1), showed ABX-type aromatic proton systems to the hydrogens H-5 [δ_H 7.01 (d, ^{3}J=8.61 Hz)], H-6 [δ_H 6.48 (dd, ^{3}J=8.35 Hz, ^{4}J=2.50 Hz)] and H-8 [δ_H 6.42 (d, ^{4}J=2.50 Hz)] and to H-3′ [δ_H 6.36 (d, ^{4}J=2.50 Hz)], H-5′ [δ_H 6.48 (dd, ^{3}J=8.35 Hz; ^{4}J=2.50 Hz)] and H-6′ [δ_H 7.01 (d, ^{3}J=8.30 Hz)]. They were assigned to ortho and meta couplings. We assigned the overlapping signals at δ_H 3.77 to the two methoxy groups. Hydrogen signals bound to oxygenated carbon were also observed at δ_H 4.35 (H-2*β*, ddd, ^{2}J=10.39 Hz, ^{3}J=3.36 Hz, ^{4}J=1.96 Hz) and δ_H 4.05 (H-2*α*, t, ^{2}J=10.10 Hz), as well as one double doublet at δ_H 2.99 (H-4*α*, dd, ^{2}J=15.78 Hz, ^{3}J=10.58 Hz) and one double doublet of doublets at δ_H 2.92 (H-4*β*, ddd, ^{2}J=15.62 Hz, ^{3}J=5.53 Hz, ^{4}J=1.79 Hz), referring to germinal-vicinal couplings. The multiplet at 3.51 ppm was assigned to H-3.

The ^{1}H-^{1}H-COSY correlations showed ortho coupling between hydrogens H-5 and H-6, and the hydrogens H-5′ and H-6′; meta couplings between hydrogens H-6 and H-8, and the hydrogens H-5′ and H-3′; geminal correlation between the hydrogens H-2*α* and H-2*β*, and axial-axial vicinal between the hydrogens H-2*α* and H-3. Same correlations were observed to the hydrogens H-4*α* and H-4*β*. Relative stereochemistry to the hydrogen H-3 was deduced based on the value of $^{3}J_{\alpha\text{-}\alpha}$ = 10.10 Hz observed in the H-2*α* signal. Furthermore, by NOESY spectrum (Nuclear Overhauser Enhancement Spectroscopy) is possible to observe correlations between several protons of the structure (Table S1). Comparing these observed correlations with the theoretical model (below) we can suggest that the structure is more compatible to the 2′-hydroxy-4′,7-dimethoxyisoflavan or (3*R*)-7-O-methylvestitol.

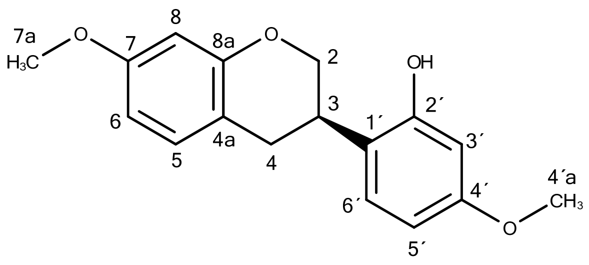

Chemistry Structure of (3R)-7-O-methylvestitol.

^{13}C-NMR spectrum (125 mHz, $CDCl_3$) displayed 12 signals corresponding to unsaturated carbon atoms in the range of 101.38 to 159.19 ppm, four of which are oxygenated (Table S1). In addition, three signals at 30.39 (C-4), 31.75 (C-3) and 69.87 ppm (C-2) were assigned to saturated carbon atoms. The signal at 55.32 ppm were assigned to the two methoxyl groups at C-7 and C-4´positions. DEPT spectrum allowed to establish the hydrogenation pattern. As shown in Table S1, ^{13}C-NMR assignments were confirmed by HSQC ($^{1}J_{H\text{-}C}$) and HMBC ($^{2}J_{H\text{-}C}$, and $^{3}J_{H\text{-}C}$, long-range correlation) correlations.

Table S1. ^{1}H-NMR, HSQC, HMBC, COSY and NOESY for $C_{17}H_{18}O_4$ (compound **3**), according to the structure described in Figure 4.

Hydrogen	δ$_H$ in ppm (multiplicity and coupling constant ^nJ in Hz)	^{1}H-^{13}C correlations		^{1}H-^{1}H correlations	
		HSQC ($^1J_{C-H}$)	HMBC ($^2J_{C-H}$ e $^3J_{C-H}$)	COSY ($^nJ_{H-H}$)	NOESY
**2*α***	4.05 (t; 10.10)	69.87 (t)	C-3, C-4, C-8a	H-2*β*, H-3	H-2*β*, H-3, H-4*β*
**2*β***	4.35 (ddd; 10.39, 3.36, 1.96)		C-3, C-4	H-2*α*, H-3	H-2*α*, H-3
**3**	3.51 (m)	31.75 (d)	C-2, C-4, C-2´, C-6´	H-2*α*, H-4*α*, H-4*β*	H-2*α*, H-2β, H-4*α*, H-4*β*, H-5
**4*α***	2.99 (dd; 15.78, 10.58)	30.39 (t)	C-2, C-3, C-4a, C-5, C-1´, C-8a	H-3, H-4*β*	H-2*α*, H-4*β*, H-6´, H-5
**4*β***	2.92 (ddd; 15.62, 5.53, 1.79)		C-2, C-3, C-4a, C-5, C-8a	H-3, H-4*α*	H-3, H-6´, H-2*α*, H-4*α*, H-5
**4a**		114.44 (s)			
**5**	7.01 (d; 8.61)	130.14 (d)	C-4, C-7, C-8a	H-6, H-4*α*	H-3, H-6, H-4*α*, H-4*β*
**6**	6.48 (dd; 8.35, 2.50)	107.11 (d)	C-4a, C-7	H-5, H-8	H-5, H-7a
**7**		159.19 (s)			
**8**	6.42 (d; 2.50)	101.38 (d)	C-6, C-7, C-4a, C-8a	H-6	H-7a
**8a**		155.35 (s)			
**1´**		119.09 (s)			
**2´**		155.14 (s)			
**3´**	6.36 (d; 2,50)	102.17 (d)	C-1´, C-2´, C-4´, C-5´	H-5´	H-4´a
**4´**		158.95 (s)			
**5´**	6.48 (dd; 8.35, 2.50)	106.08 (d)	C-1´, C-3´	H-3´, H-6´	H-4´a, H-6´
**6´**	6.98 (d; 8.30)	128.20 (d)	C-3, C-2´, C-4´	H-5´	H-4*α*, H-4*β*, H-5´
**-OCH$_3$ (7a)**	3.77 (s)	55.32 (q)	C-7		H-6, H-8
**-OCH$_3$ (4´a)**	3.77 (s)	55.32 (q)	C-4´		H-3´, H-5´
**OH**	5.04 (s)				

s= singlet; m=multiplet; d= doublet; dd= double doublet; ddd= double doublet of doublets; t= triplet; q= quadruplet. Chemical shift in ppm; Coupling constant (^nJ) em Hz. Carbon mutiplicity were defined by DEPT
